# The Decay of Surgery

**Published:** 1894-06-16

**Authors:** 


### The First Century of English Medical Literature.

### V.—THE DECAY OF SURGERY.

The art of surgery was at a very low ebb in England in the middle of the sixteenth century. The Royal College of Surgeons, founded in 1460, united itself in 1540 with the Company of Barbers under the name of the "Masters, Governors, and Commonality of the Art and Science of Surgery in London." They undertook the examination of candidates for licences to practise surgery; and by an enactment of Henry VIII. all persons were prohibited from practice unless such a licence had been first obtained. But the standard must have been a curious one, to judge from the complaints with which the literature of the times is laden. Two causes seem principally to have occasioned the evils deplored by all. The first was the low esteem in which the art of surgery was held by the learned; this popular contempt for the profession not only hindered able men from adopting it, but deterred many from resorting to surgical aid even when most needed. The prejudice was not overcome until the translation of the works of learned surgeons in Italy, France, and Germany awoke a new zeal in English practitioners, and induced skilled physicians to devote themselves to the kindred art. The second cause of the decay of surgery was a natural consequence of the first, viz., the vast number of unskilled practitioners whose ignorance and cruelty brought the whole art into disrepute.

One of the earliest who attempted to raise English surgery to a higher level was John Hall, a member of the Company of Surgeons, and long in practice as a surgeon at Maidstone in Kent. He is honourably distinguished as a bitter enemy to magic and quackery, against which he wrote an historical expostulation of no small interest. In 1564 he published a translation of the "Chirurgia Parva" of Lanfrancus, and from his dedicatory letter to the whole company and brotherhood of the chirurgeons of London much light is thrown on the condition of the art at that date. "I cannot but a little marvel," he writes, "at the stubborn, disdainful ignorance of many, in this our wretched age, wherein the noble art of chirurgery is fallen, as it were, into ruin. . . . The despisers thereof ofttimes, as we see, through hate that they bear to these excellent parts and the ministers thereof by negligence, to run to extreme desolation; and therefore many times worthily perish as a just reward for their contempt of God, His ordained remedies." But it can hardly be matter for surprise that many shrank from the surgeon's good offices when we read later on: "Whereas there is one in England, almost throughout all the realm, that is indeed a true minister of this art, there are ten abominable abusers of the same. . . . Yea, smiths, cutlers, carters, coblers, coopers, curriers of leather, carpenters, and a great rabble of women. Which forsake their handycrafts and for filthy lucre abuse physic and surgery." Some form of instruction appears to have been available for the student, but Hall complains, "There are many that take servants and apprentices not for to teach them science, but only to be their drudge and to do their toil and labour, which is the cause that so many come out of their years so ignorant; yea, and some will refuse a young man that is learned and apt to understand, and have an ignorant slave to bear the water tankard and scour pans."

In translating the works of Laufrancus, Hall mentions other very ancient copies of it in English to which he has had access, notably one in Anglo-Saxon dating back two hundred years. Laufrancus, who came to Paris from Milan towards the end of the thirteenth century, was the first to revive the art of surgery in France. He comments in terms almost identical with those of Hall on the miserable state of French surgery in his day, complaining that the surgeons were nearly all idiots (scarcely knowing their own language), all laymen, true quacks, and so ignorant that it was scarcely possible to find a rational chirurgeon. He was himself a disciple and imitator of Guillaume de Salicet, and is principally noteworthy for his condemnation of the system of employing tents in the treatment of wounds, which survived, however, for many centuries after.

Further details concerning the low condition of surgery in England are found in epistles by John Banister, prefixed to a treatise of surgery which he translated in 1575, from Calmetus and Tagalt. He speaks of the art "as rather despised as needless than regarded as necessary," and lays it to the account of the unqualified practitioners, over whom he exhausts his powers of invective, not sparing the College of Surgeons itself. "Very hard it were for such a rout of roguish livers so boldly to practice and to fill all places with the slanderous shavings of their devilish practises, were they not permitted of some in authority, and winked at of them that should them punish. I do not a little marvel also how, and by what means or merit, so many get their authorities and licence to practise, when, as in examination, they not only show themselves ignorant asses, and in their works bussardly begards, but also of themselves disposed to daily dissimulation, without either fear of God or obedienee to their Prince; I, myself, know them at this day the most beastly deceivers and licentious livers that ever professed any art, having neither knowledge, modesty, nor honesty, and yet practise their accustomed deceits under the colour of admittance from our Hall, being, indeed, far fitter for the cart than chirurgery."

It is not to be supposed that there were not some good surgeons among the Fellows of the College. Banister himself was a learned and skilful practitioner, having won his professional experience in the best of all fields for the purpose at that time, viz.: the field of battle. By the aid of a royal letter of recommendation, he obtained licence from the College of Physicians to practise physic in addition to surgery, and settled in London, where he gained much renown, and is celebrated not less for his warm friendship with members of his profession, than for his generosity to the poor, and especially to old soldiers. He seems to have formed a centre round which all the newly awakening professional zeal had its rise. George Baker, surgeon to Queen Elizabeth, who has been already mentioned as the translator of Gesner, was among his intimates, as were also Balthrop, William Clowes, an early friend to whom he was always faithful, Goodrus, Read, and Thomas Vicary, of St. Bartholomew's Hospital. It is owing to this little cluster of friends that some of the grosser abuses of the craft were slowly repressed, and the means of knowledge were placed within reach, at any rate, of those willing to be taught.

